# A Meta-Analysis and Systematic Review on the Association between Human Papillomavirus (Types 16 and 18) Infection and Esophageal Cancer Worldwide

**DOI:** 10.1371/journal.pone.0159140

**Published:** 2016-07-13

**Authors:** Jing Wang, Lei Zhao, Han Yan, Juanjuan Che, Li Huihui, Wu Jun, Bing Liu, Bangwei Cao

**Affiliations:** 1 Department of Oncology, Beijing Friendship Hospital, Capital Medical University, Beijing, China; 2 Department of Emergency, Beijing Friendship Hospital, Capital Medical University, Beijing, China; State University of Maringá/Universidade Estadual de Maringá, BRAZIL

## Abstract

**Background:**

Esophageal cancer is a common and aggressive malignant tumor. This study aimed to investigate the association between human papillomavirus (HPV) Types 16 and 18 and esophageal carcinoma (EC) in the world population by conducting a meta-analysis.

**Materials and Methods:**

Computerized bibliographic and manual searches were performed to identify all eligible literatures between 1982 and 2014. PUBMED (http://www.ncbi.nlm.nih.gov/pubmed/) and CNKI (http://www.cnki.net/) were the primary sources of case-control studies, and key words used include human papillomavirus, HPV, esophageal, esophagus, cancer, carcinoma, and tumor. All searches were performed by reviewing articles and abstracts cited in the published systematic reviews and case-control studies. Prospective studies that reported relative risk (RR) estimates with 95% CIs for the association between HPV and EC were included.

**Results:**

Thirty-three randomized studies were identified, and the main features of these trials were included in this systematic review. HPV infection rate in the EC group was 46.5%, while HPV infection rate in the control group was 26.2% (OR = 1.62; 95% CI, 1.33–1.98). In China, the merger OR value was 1.62 (95% CI: 1.26–2.07); while in the Asian region, the merger OR value was 1.63 (95% CI: 1.29–2.04). There were statistical differences in HPV testing due to different detection methods such as PCR, IHC and ISH. In the PCR detection group, the merger OR value was 1.61 (95% CI: 1.33–1.95).

**Conclusions:**

These results indicate that HPV infection and the incidence of EC are closely associated.

## Introduction

Esophageal carcinoma (EC) is the most aggressive malignant tumor of the gastrointestinal tract and the eighth most commonly occurring cancer in the world [1]. It has been well recognized that the development of EC involves multiple factors in a multistage process [2]. Alcohol, tobacco, nutritional deficiencies, infectious agents, etc. were confirmed to have a relationship to esophageal carcinogenesis [3]. However, many physical, chemical and biological factors related to EC remain unknown.

Human papillomavirus (HPV) infections, especially high-risk types 16 and 18, have recently been reported as a possible risk factor for EC. However, direct evidence of this relationship has been lacking, and results of those studies were not consistent. This study aimed to conduct a meta-analysis, and systematic review of literature to determine whether an association exists between HPV type 16 and 18 infection and EC.

## Materials and Methods

### Data Sources

A literature search was performed from 1982 to 2014 using PUBMED and CNKI databases without restrictions, and the following search terms were used: (human papillomavirus, HPV) and (esophageal, esophagus) and (cancer, carcinoma, tumor). Moreover, reference lists were reviewed to search for relevant studies. This systematic review was planned and reported in adherence to the standards of quality for reporting systematic reviews.

### Inclusion and Validity Criteria

All searches were performed by reviewing articles and abstracts cited in the published systematic reviews and case-control studies. Inclusion criteria were as follows: (1) prospective case-control studies, (2) EC diagnosed by pathology, (3) Control group obtained from esophageal epithelial tissues of normal individuals (screening) or normal marginal tissues of EC.

### Exclusion criteria

Exclusion criteria included the following: reports of poor quality, duplicate reports, inadequate information, unclear data descriptions or samples were removed, sample size less than 20, and abstracts.

### Quality evaluation

The Jadad scoring method was used to evaluate several aspects of the quality of the study. Three independent researchers extracted data, blindly evaluated the quality of the literature, and analyzed the data including withdrawals and dropouts [4]. If there were differences of opinion, the cases were discussed until consensus was achieved.

### Statistical Analysis

A heterogeneity test was used to select the method of data combination in the systematic review. The Cochrane’s Q-test was performed and I^2^ statistics were obtained, using a predefined significance threshold of 0.05.If *P*≥0.05, it was considered that there was no heterogeneity between studies; and a fixed effects model (FEM) was used for the analysis. If *P*<0.05, it was considered that heterogeneity existed between studies; and a random effects model (REM) was used after correction for analysis. If a null hypothesis, which represents that there was no heterogeneity among each study, was accepted, the Mantel-Haenszel fixed-effects model was used to calculate the combined odds ratio (OR) with 95% confidence intervals (CI) and the Forest Plot. If the null hypothesis was rejected, REM was used to calculate the combined OR with 95% CI and the Forest Plot. To detect publication bias, the asymmetry of standard error–based funnel plots was examined using the linear regression method, as suggested by Egger *et al*. [5]. The Stata10.0 software (Stata Corporation, College Station, Texas) was used for the statistical analysis in this study.

### Subgroup analyses

EC incidence in subgroups were stratified and analyzed according to various geographical areas, the control group selection method, and various HPV detection methods.

## Results

### Search Results

**Fig 1:**
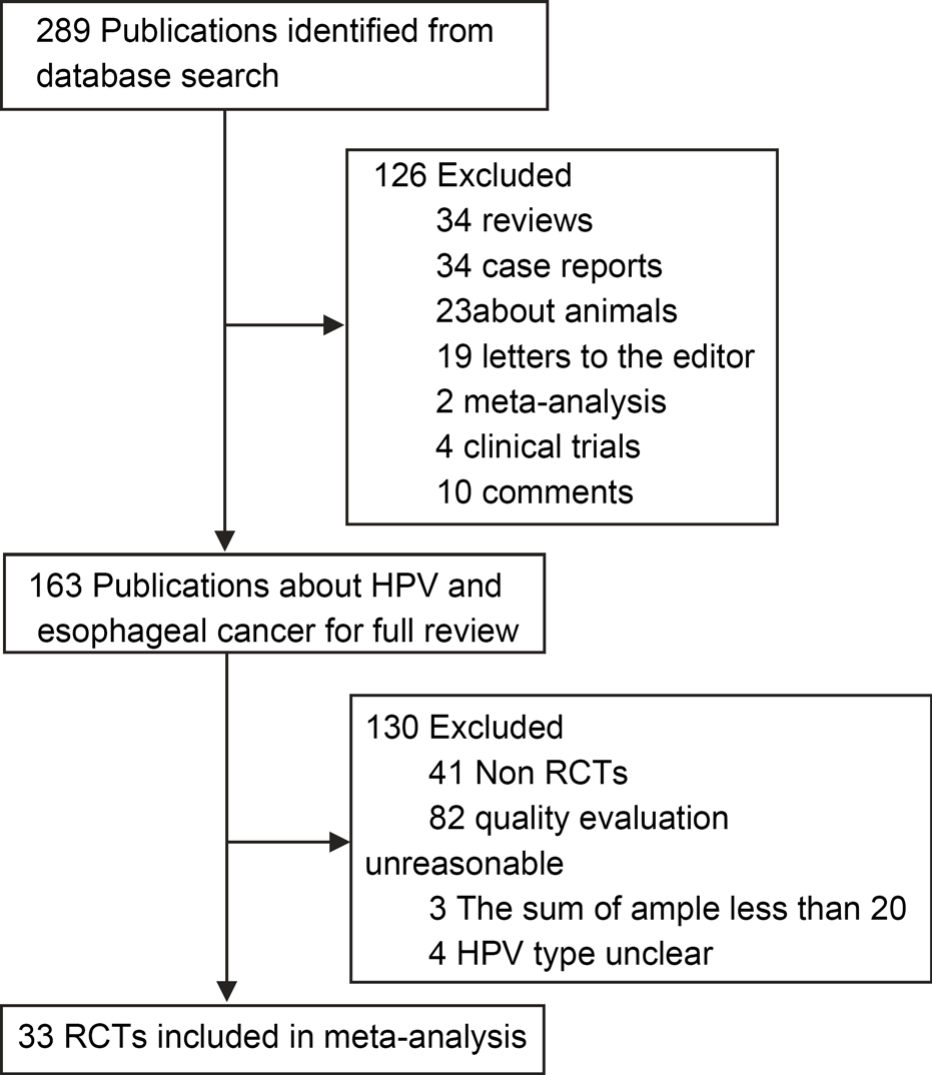
Selection of studies for inclusion in the meta-analysis.

A total of 297 articles were identified by using the search criteria, and these studies were carried out from 1982 to 2014. All studies were obtained from published literature (Fig 1). Nine countries including China, Iran, Italy, Greece, Egypt, Sweden, Japan, France and Mexico were involved in the case-control studies. A total of 33 case-control studies were selected for analysis. There was no statistical significance in factors such as gender or age between the two groups. The PRISMA checklist is shown in S1 PRISMA Checklist.

### Information of articles

Detailed steps of the literature search are shown in Fig 1. The inclusion and exclusion of case-control studies for this systematic review are shown in the flow chart. Briefly, 33 articles matched the standard [6–38]. Among the 2,430 cases in the EC group, 1,131 were HPV positive (46.54%); while among the 3,621 cases in the control group, 977 were HPV positive (26.98%). In the control group, 14 samples came from adjacent normal tissues of gastrointestinal cancers, while 19 samples came from normal esophageal specimens. These samples were obtained during the esophageal cancer screening of healthy people living in areas of high incidence of esophageal cancer (Table 1).

**Table 1:** Characteristics of the 33 case-control studies included in the meta-analysis.

Literature sources	HPV(+)/	HPV(+)/	Area	Incidence	Detection Method	Control selection	HPV types
	cases group	control group					
Li Y, 1991	12/24	9/24	China	Unclear	ISH	Adjacent-normal	16
Han C, 1996	22/90	6/121	China	High	ELISA	True-normal	16
Ren ZHP, 1996	35/52	2/30	China	Lower	IHC	True-normal	16,18
Wang XJ, 1998	20/40	36/58	China	High	ISH	True-normal	16
Lu LC, 1999	15/55	43/55	China	High	ISH	Adjacent-normal	18
Cao BW 2005	207/265	203/357	China	High	PCR	True-normal	16, 18
Liu J, 2000	44/60	23/56	China	High	IHC	True-normal	16,18
Yu DH, 2000	78/112	42520	China	Lower	IHC	True-normal	16,18
Kuang ZS, 2000	23/56	4/56	China	High	PCR	Adjacent-normal	16,18
Sun LB, 2001	42542	42390	China	Lower	PCR-RT	Adjacent-normal	16
Shen ZY, 2002	115/176	105/176	China	High	PCR	Adjacent-normal	6, 11, 16, 18
Xu WG, 2003	28/40	10/50	China	Unclear	ISH	True-normal	16
Zhou XB, 2003	31/48	42605	China	High	PCR	Adjacent-normal	16
Xu CL, 2004	16/18	126/183	China	Lower	IHC	Adjacent-normal	16
Lu HS, 2004	3/40	13/39	China	Lower	IHC	Adjacent-normal	16
Lu XM, 2004	55/104	41/104	China	High	PCR	Adjacent-normal	16
Chen J, 2004	14/30	7/60	China	Lower	PCR	True-normal	16
Jiang HY, 2005	48/65	11/65	China	Lower	IHC	Adjacent-normal	16,18
Kamangar F, 2006	33/99	106/381	China	High	ELISA	True-normal	16,18,73
Zhou SM, 2009	26/82	10/80	China	High	PCR	Adjacent-normal	16
Guo F,2012	93/300	61/900	China	High	PCR	True-normal	16,18,58
Bahnassy AA, 2005	27/50	12/50	Egypt	Unclear	PCR	Adjacent-normal	16,18,11
Benamouzig R, 1992	42502	42393	France	Unclear	ISH	True-normal	6, 11, 16, 18,31, 33,
Lyronis, 2008	17/30	42548	Greece	Unclear	PCR	True-normal	16,18
Farhadi M, 2005	14/38	5/38	Iran	High	PCR	True-normal	L1,18
Far AE, 2007	33/140	12/140	Iranian	Unclear	PCR	Adjacent-normal	16.18.31.33
Astori G, 2001	42568	42476	Italy	High	PCR	Adjacent-normal	16
Tornesello ML, 2009	12/56	42609	Italy	Unclear	PCR	True-normal	6,16,8, 15, 20,25
Khurshid A, 1998	17/27	42441	Japan	Unclear	PCR	True-normal	16,18,33
Kawaguchi H, 2000	42721	25/58	Japan and China	Unclear	PCR	True-normal	16/18
Dąbrowski A, 2012	28/56	4/35	Lublin	Unclear	PCR	True-normal	16,18
Acevedo, NE, 2004	15/17	42544	México	Unclear	PCR	True-normal	16,11
Lagergren J, 1999	20/193	61/302	Sweden	Unclear	ELISA	True-normal	16,18

Lower: lower incidence of esophageal carcinoma (EC); Higher: higher incidence of EC; PCR: polymerase chain reaction; IHC: immunohistochemistry; ISH: in situ hybridization; ELISA: enzyme-linked immunosorbent assay. Adjacent-normal: the control group was obtained from the normal marginal tissue of EC during surgery. True-normal: controls were obtained from the normal esophageal epithelial tissue.

### Heterogeneity test

Thirty-three tests for heterogeneity (*i* = 94.78, *P*<0.001) revealed that there was heterogeneity between studies and between subgroups. Therefore, overall and subgroup analyses were corrected using REM; and the method of DerSimonian-Laird as used to merge data, and calculate ORs and 95% CIs. Egger’s regression analysis was used to more objectively evaluate publication bias (Table 2).

**Table 2:** Quality assessment and subgroup analysis of HPV infection and esophageal carcinoma.

Group	No. of Studies	Heterogeneity of ORs			Model used	Egger's test		
		*χ2* (df)	*P*	OR (95% CI)		*χ2*	*P*	95% CI
ALL	33	78.4	<0.001	1.45 (1.34–1.57)	REM	2.05	0.058	-0.01–2.64
Area								
High	13	31.93 (11)	<0.001	2.96 (2.01–4.34)	REM	1.71	0.173	-0.61–3.03
Lower	7	56.21 (7)	<0.001	3.57 (1.15–11.12)	REM	0.37	0.443	-2.90–5.68
China	21	31.93 (12)	<0.001	2.96 (2.01–4.35)	REM	1.69	0.130	-0.46–3.33
Non-China	12	56.21 (8)	<0.001	3.57 (1.15–11.13)	REM	0.37	0.558	-4.35–2.49
Asia	24	31.93 (13)	<0.001	2.96 (2.01–4.36)	REM	1.73	0.090	-0.24–0.017
Non-Asia	9	56.21 (9)	<0.001	3.57 (1.15–11.14)	REM	0.37	0.091	-0.67–7.04
Method								
PCR	18	20.39 (10)	0.030	3.49 (2.49–4.90)	REM	1.25	0.012	0.91–2.38
IHC	6	38.43 (5)	<0.001	4.76 (1.36–16.63)	REM	0	0.659	-5.02–7.10
ELSIA	4	20.02 (2)	<0.001	0.90 (0.13–6.48)	REM	0	0.666	-74.37–81.47
ISH	5	19.03(2)	<0.001	0.90 (0.15–6.42)	REM	0	0.254	-4.30–11.12
Self								
Self	14	56.09 (9)	<0.001	2.18 (1.02–4.66)	REM	0.89	0.200	-0.80–3.41
NO	19	36.69 (9)	<0.001	4.58 (2.69–7.80)	REM	2.33	0.102	-0.32–3.22

PCR: polymerase chain reaction; IHC: immunohistochemistry; ELISA: enzyme-linked immunosorbent assay; ISH: in situ hybridization; REM: random effects model; OR: odds ratio; CI: confidence interval.

### The relationship of HPV types 16 and 18 and EC

**Fig 2:**
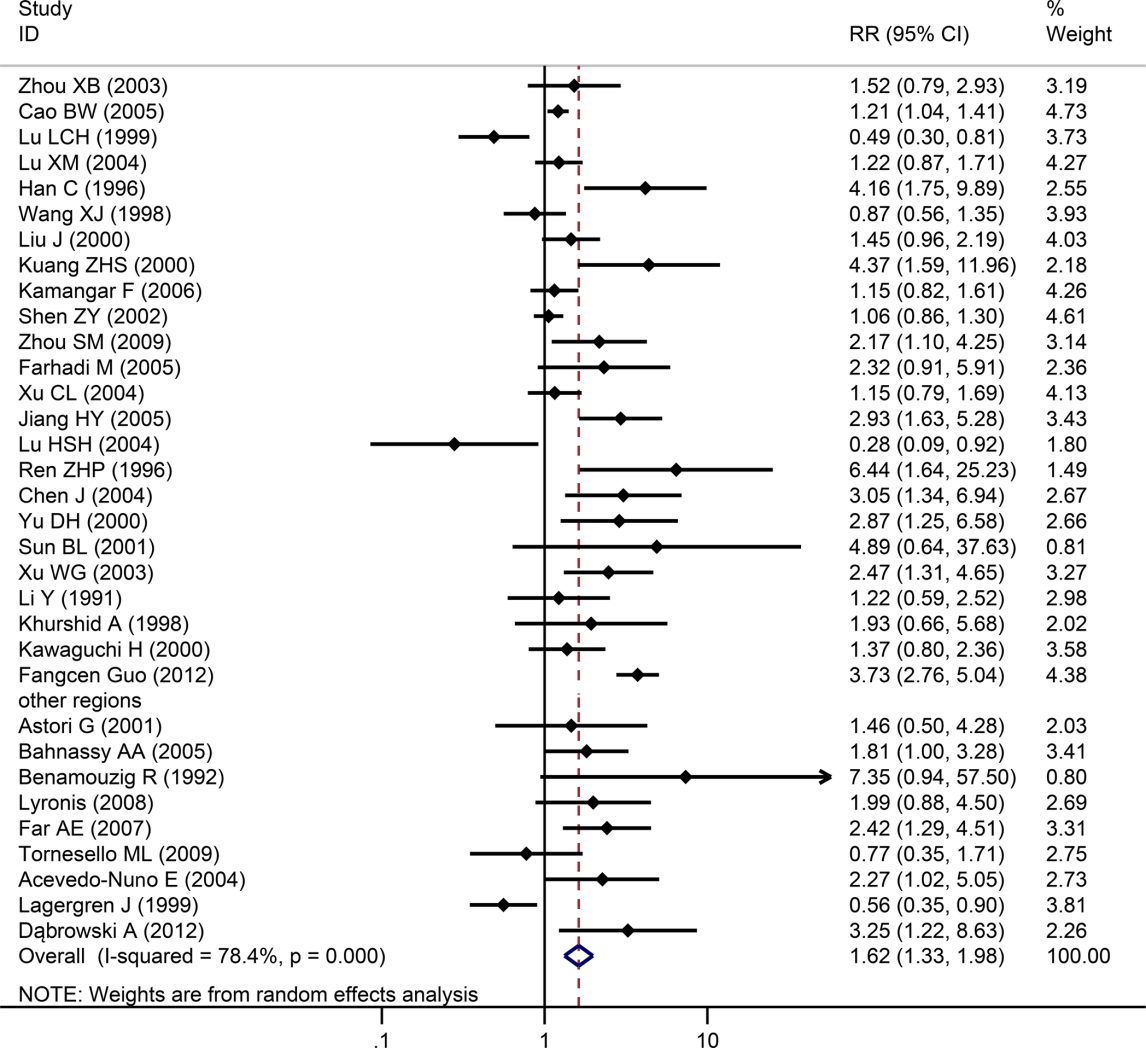
Individual trial and overall risk ratios of the association between HPV (types 16 and 18) infection and esophageal carcinoma.

**Fig 3:**
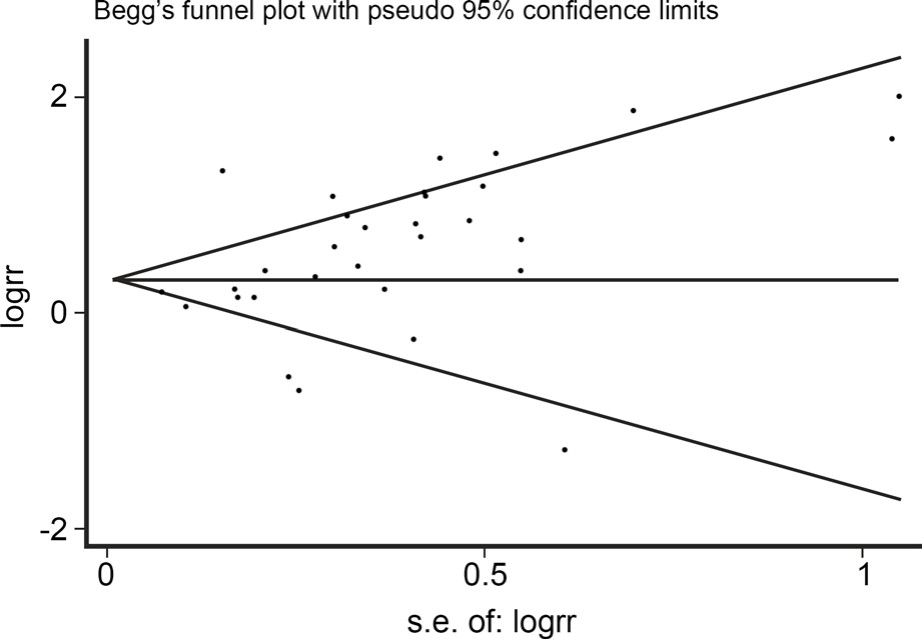
Begg’s funnel plot on studies of HPV infection and esophageal carcinoma.

Among the 33 studies, infection rate in the EC group was 46.5%, while infection rate in the control group was 27.0%. Heterogeneity test revealed that there was heterogeneity (*χ*^*2*^ = 78.4, *P*<0.001) with a *P*<0.05 for the *Q*-test. A REM analysis was applied. The combined effect of these test results revealed an association between HPV infection and EC. From the independent OR and synthetic OR of the 33 researches, HPV infection was found to be closely associated with EC. The merger OR value was 1.62; 95% CI of 1.33–1.981.57, Fig 2. The accuracy of each study as argument, with OR/SE as the dependent variable, had a 95% CI of -0.0111784–2.63736 (*P* = 0.058). Therefore, there was no significant bias in the publications, there was no substantive effect on the synthetic OR, and the conclusion was reliable (Fig 3).

### Subgroup systematic review

**Fig 4:**
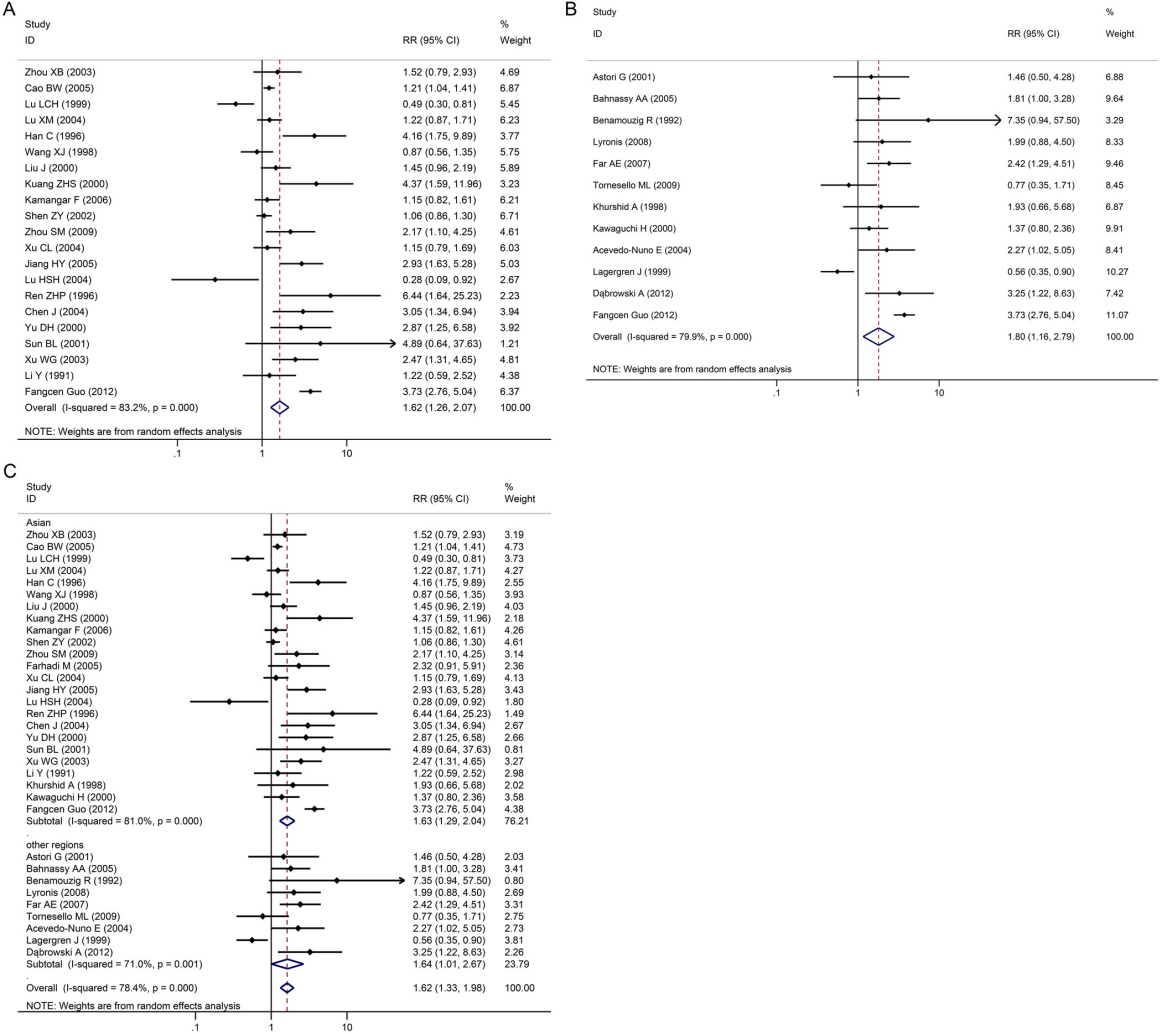
Individual trial and overall risk ratios of relationships between HPV infection and esophageal carcinoma in various geographical areas. 4A: China, 4B: non-China and 4C: Asia/non-Asia.

**Fig 5:**
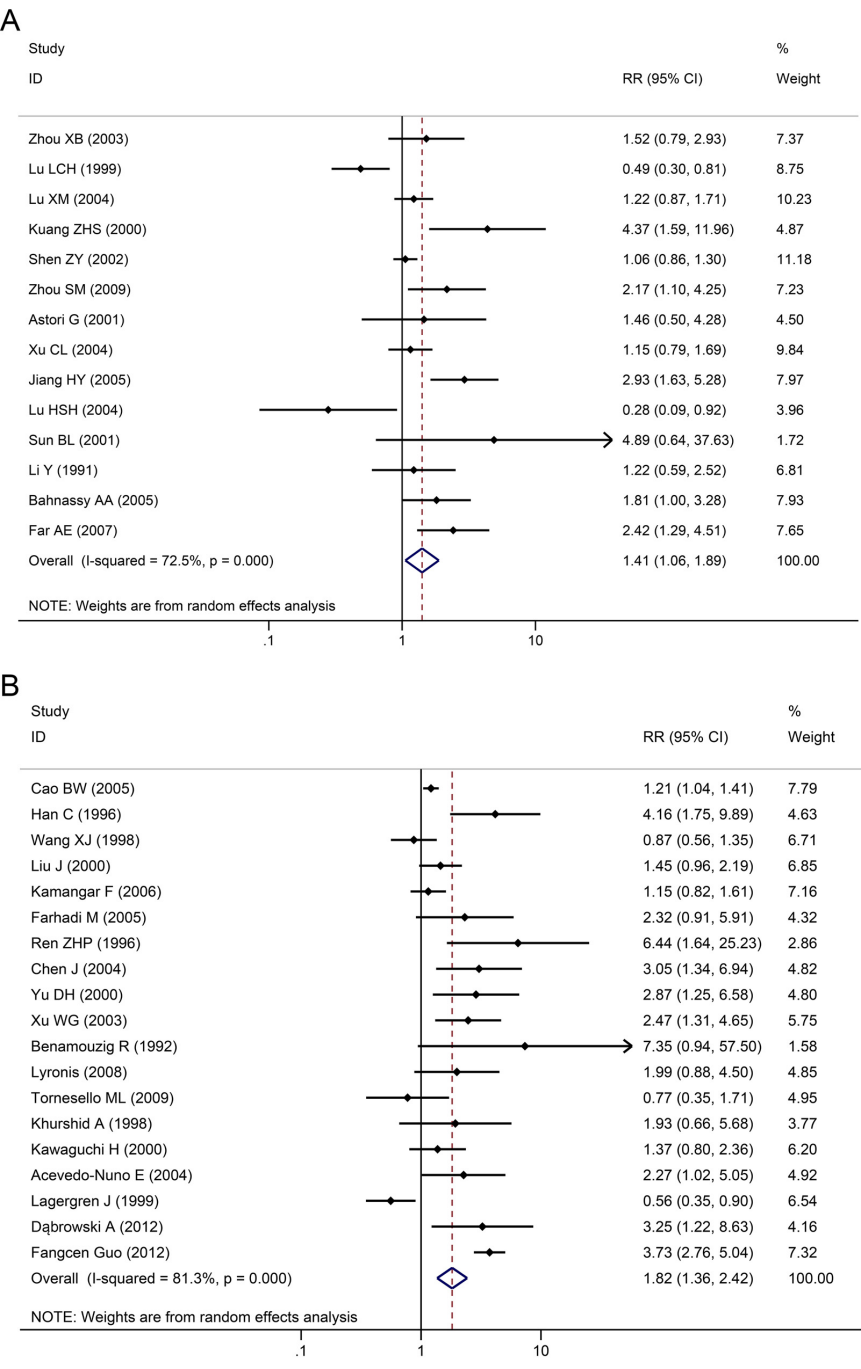
Individual trial and overall risk ratios of relationships between HPV infection and esophageal carcinoma compared to various controls.

EC incidence in subgroups were stratified and analyzed according to various geographical areas (Fig 4A–4C), the control group selection method (Fig 5), and various HPV detection methods.

There were 21 case-control studies in China. The merger OR value was 1.62 (95% CI: 1.26–2.07) including high incidence areas for EC such as the Taihang, Qinling and Dabie mountain ranges, as well as the northeast of Sichuan, east of Xinjiang, east of Fujian and east of Guangdong provinces. No publication bias (Egger’s test, *P*>0.05) was found, indicating that the results had good reliability. In other regions (outside China) such as Egypt, France, Greece, Iran and other countries, merger OR value was 1.80 (95% CI: 1.16–2.79); and there was also no publication bias (Egger’s test, *P*>0.05). In Asian countries such as China, Japan and India, the merger OR value was 1.63 (95% CI: 1.29–2.04); and there was no publication bias (Egger’s test, *P*>0.05). In non-Asian countries such as Egypt, France, Greece, Iran, Italy, Mexico and Sweden, merger OR value was 1.64 (95% CI: 1.01–2.67); and there was no publication bias (Egger’s test, *P*>0.05). In addition, results were same in both high risk and low risk areas. In high risk areas, merger OR value was 1.29 (95% CI: 1.05–1.59); while in low risk areas, merger OR value was 2.06 (95% CI: 1.08–3.94). (Fig 4A–4C)

In the control patient group (healthy individuals), merger OR value was 1.41 (95% CI: 1.06–1.89); while in the control tissue group (normal tissues from EC patients), merger OR value was 1.82 (95% CI: 1.36–2.42, Fig 5).

**Fig 6:**
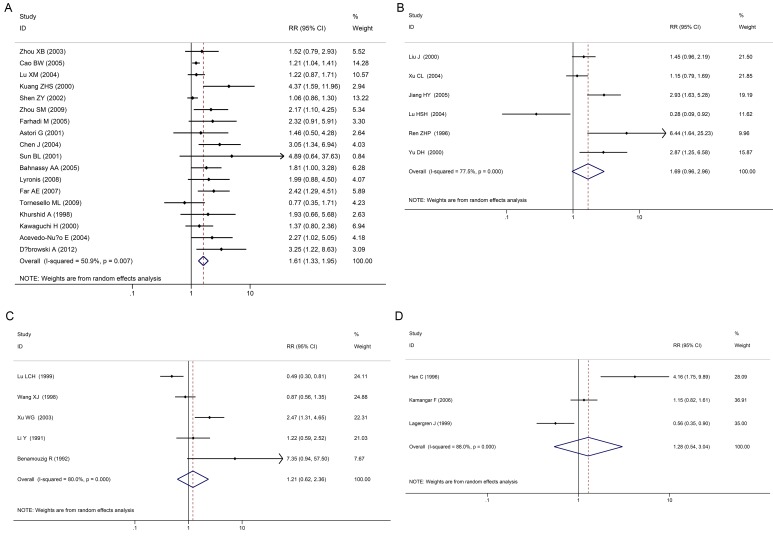
Individual Trial and Overall Risk Ratios of Relationships between HPV Infection and EC using Various Detection Methods. (6A:PCR, 6B IHC, 6C:ISH, 6D:ELISA).

Various HPV detection methods were summarized and analyzed: 18 cases were detected by PCR, 6 cases were detected by ISH, 6 cases were detected by IHC, and 3 cases were detected by ELISA. In the PCR group, merger OR value was 1.61 (95% CI: 1.33–1.95); in the ISH group, merger OR value was 1.21 (95% CI: 0.62–2.36); and in the IHC group, merger OR value was 1.69 (95% CI: 0.96–2.96). An opposite result was acquired in the ELISA group, where the merger OR value was 1.28 (95% CI: 0.54–3.04; Fig 6A–6D).

## Discussion

EC is the fifth most common cancer in developing countries, and the eighth most common cancer worldwide[39]. Areas of high prevalence for EC are mainly located in developing countries, and there are obvious regional differences. [40] The world’s highest areas of incidence are located in Asia, which is called, the "EC belt" [41]. ECs vary greatly by geographic distribution, in which there is a higher incidence in China, America and the eastern Himalayas [42]. However, the incidence of esophageal cancer is low in developed Western countries.

There are several proposed risk factors for EC including eating habits, tobacco, alcohol, pollution, genetic factors, infection of HPV viruses and EBV (Epstein-Barr) virus [43], family history (immediate blood relatives within three generations), etc. In developed countries, tobacco and alcohol is a major factor [42–44]. However, it is different in countries with high incidence of EC, as few cases are attributed to smoking or alcohol consumption there [45].

Currently, the role of HPV infection in esophageal cancer is unclear. Many studies from Africa and China have shown that HPV infections were associated with esophageal cancer. However, in areas with lower prevalence of HPV, there was no decrease in risk of EC [46–49]. The differences in the results of the studies may be due to: 1. Differences in race, living habits, environmental factors that lead to HPV infection. 2. Differences in research design, detection means, methods, statistical analysis. The aim of this study was to perform a comprehensive analysis of the data to determine the relationship between EC and HPV.

In order to explain the association between HPV and EC, we reviewed many articles and selected only case-control studies for analysis. To our knowledge, this is the first study that conducted a systematic review of the relationship between HPV infection (types 16 and 18) and EC worldwide. Results of the comprehensive evaluation of this final systematic review revealed that there was a significant association between HPV infection and EC risk with an integrated OR = 1.62 (95% CI: 1.33–1.98). In META analysis, the presence or absence of heterogeneity directly affected the results of the statistics. Therefore, this study was conducted to strictly evaluate heterogeneity.

HPV infection rates may be related to geographical location. Therefore, we preformed a subgroup analysis according to geographical location worldwide. The stratification study revealed that regardless of whether the studies were in China, Asia, outside of China, or outside of Asia, or in high or low risk areas, HPV infection was associated with EC. In the current research, most studies from low-risk areas also had an association between HPV and EC, and these correlations were stronger than high-risk areas; which was different from other reports. A possible reason was that in high-risk areas, EC occurred due to other reasons such as eating habits, tobacco, alcohol, pollution, genetic factors and HPV infection; thus, the influence of HPV decreased [50]. In low-risk areas, HPV infection appears to be the main cause.

Although most of the esophageal cancer risk factors have been determined, there may still be unknown confounders that may interfere with the results. The literature included case-control studies that were inevitably affected by a variety of bias.

However, due to the current study design, many kinds of bias were possible. (1) Most studies detected HPV from tumor tissues and tissues around tumors that could have been infected with HPV. (2) This systematic review included many studies from all over the world. The variable HPV prevalence could have been related to the various geographic regions studied.

The choice of the control group may have also affected the results, because the healthy controls group revealed that HPV was associated more closely with EC. Cancer adjacent tissues as controls also confirmed a link between HPV and EC, but with a weaker correlation than the cancer tissue itself. This may be related to the HPV infection of the cancer tissue in the body or contamination of samples. There are many methods for detecting HPV, and there was no uniform detection method used in these studies. The researchers did not make use the same HPV detection methods used such as PCR, ELISA, ISH and IHC; although some of the early HPV detection techniques have since been abandoned. Therefore, these results are likely to have some bias, and PCR seems to be the most accurate monitoring method [51].

The current result was the same as that reported by Zhang et al. [48,52] except that their study was limited to a Chinese population, and used a PCR detection method for HPV16. Zhang et al. found that there was a relatively high level of HPV 16 prevalence in Chinese patients with esophageal cancer, and concluded that HPV-16 infection may be a risk factor for esophageal cancer. The current research included the entire world population, and all methods to detect HPV16. Using a subgroup analysis, we obtained results similar to those of Zhang et al. While, there was the limitation that the analyses did not distinguish between the two primary histologic forms of esophageal cancer: squamous cell carcinoma and adenocarcinoma. From our research, squamous cell carcinoma were more likely linked to HPV, adenocarcinoma was fewer than squamous cell carcinoma. This may be related to its biological characteristics.

## Conclusions

These results indicate that HPV is closely associated with EC in China, Asia, and all over the world. In investigating the association between HPV and EC, the selection of the control group is important in order to avoid interfering factors. This systematic review provides epidemiologic evidence to support the association between HPV infection and EC. Multi-center studies on regional incidences with strict control of false positives and false negatives would be needed to confirm the association of HPV and EC. If confirmed, HPV testing may be useful in groups at high risk for EC; while HPV vaccine might be useful as a primary prevention measure.

**Guarantor of the article:** Bangwei Cao, PhD and Bing Liu

## Supporting Information

S1 PRISMA Checklist The PRISMA checklist. (DOC)

## Abbreviations

HPV: human papillomavirus
EC: esophageal carcinoma
RR: risk ratio
CIs: confidence intervals
PCR: polymerase chain reaction
ISH: *in situ* hybridization
ICH: immunohistochemistry
ELISA: enzyme-linked immunosorbent assay
FEM: fixed effects model
REM: random effects model
OR: odds ratio
EBV: Epstein-Barr
